# Hypothesis: Pulmonary Afferent Activity Patterns During Slow, Deep Breathing Contribute to the Neural Induction of Physiological Relaxation

**DOI:** 10.3389/fphys.2019.01176

**Published:** 2019-09-13

**Authors:** Donald J. Noble, Shawn Hochman

**Affiliations:** Department of Physiology, Emory University School of Medicine, Atlanta, GA, United States

**Keywords:** slow, deep breathing, relaxation, neurophysiological mechanisms, slowly-adapting pulmonary afferents, slow brain rhythms

## Abstract

Control of respiration provides a powerful voluntary portal to entrain and modulate central autonomic networks. Slowing and deepening breathing as a relaxation technique has shown promise in a variety of cardiorespiratory and stress-related disorders, but few studies have investigated the physiological mechanisms conferring its benefits. Recent evidence suggests that breathing at a frequency near 0.1 Hz (6 breaths per minute) promotes behavioral relaxation and baroreflex resonance effects that maximize heart rate variability. Breathing around this frequency appears to elicit resonant and coherent features in neuro-mechanical interactions that optimize physiological function. Here we explore the neurophysiology of slow, deep breathing and propose that coincident features of respiratory and baroreceptor afferent activity cycling at 0.1 Hz entrain central autonomic networks. An important role is assigned to the preferential recruitment of slowly-adapting pulmonary afferents (SARs) during prolonged inhalations. These afferents project to discrete areas in the brainstem within the nucleus of the solitary tract (NTS) and initiate inhibitory actions on downstream targets. Conversely, deep exhalations terminate SAR activity and activate arterial baroreceptors via increases in blood pressure to stimulate, through NTS projections, parasympathetic outflow to the heart. Reciprocal SAR and baroreceptor afferent-evoked actions combine to enhance sympathetic activity during inhalation and parasympathetic activity during exhalation, respectively. This leads to pronounced heart rate variability in phase with the respiratory cycle (respiratory sinus arrhythmia) and improved ventilation-perfusion matching. NTS relay neurons project extensively to areas of the central autonomic network to encode important features of the breathing pattern that may modulate anxiety, arousal, and attention. In our model, pronounced respiratory rhythms during slow, deep breathing also support expression of slow cortical rhythms to induce a functional state of alert relaxation, and, via nasal respiration-based actions on olfactory signaling, recruit hippocampal pathways to boost memory consolidation. Collectively, we assert that the neurophysiological processes recruited during slow, deep breathing enhance the cognitive and behavioral therapeutic outcomes obtained through various mind-body practices. Future studies are required to better understand the physio-behavioral processes involved, including in animal models that control for confounding factors such as expectancy biases.

## Introduction

Respiration is a regulable variable under voluntary control with access to central autonomic circuits linked to the modulation of cognition and behavior. Relaxation techniques involving slow, deep breathing have shown promise in a variety of cardiorespiratory and stress-related disorders including chronic heart failure, hypertension, anxiety, and depression (Brown and Gerbarg, 2005; Jerath et al., 2006). Despite an abundance of mind-body practices that involve slow, deep respiration as a fundamental component, our knowledge of the optimal parameters and underlying mechanisms of deep breathing for therapeutic benefit remains incomplete. Our own work (Noble, 2015) and several recent reviews (Russo et al., 2017; Zaccaro et al., 2018) have sought to synthesize research on the psychophysiology of slow breathing, highlighting the impressive degree of cardiorespiratory synchrony resulting from these practices. Here we explore how slow breathing may modify cognition by promoting relaxation (reduced stress reactivity) and alert functional states (enhanced attention and memory consolidation). Expanding on the recent identification of brainstem neurons linked to reductions in behavioral arousal (Yackle et al., 2017; Chen et al., 2018), we emphasize respiratory-based changes in afferent encoding that include recruitment of pulmonary slowly-adapting mechanoreceptors and their putative interactions with central autonomic circuitry (Jerath et al., 2006). We also describe olfactory-hippocampal pathways that may mediate the selective effects of slow nasal breathing on memory function.

Our neurophysiological model for the mechanisms mediating effects of slow, deep breathing proposes a central role for vagal myelinated lung afferent pathways through brainstem relay nuclei. It is important to keep in mind that these pathways do not function in isolation but instead are integrated within a larger network of sensory and motor circuits that contribute to the overall cognitive experience of slow breathing, including descending motor drive from higher-level brain centers (e.g., Del Negro et al., 2018). Voluntary slow breathing may engage respiratory motor networks, since an individual can sense the motor effort required to inhale larger volumes. However, there is also evidence that voluntary breathing might not be essential to realize the basic physiological benefits of these techniques; spontaneous slow breathing without cognitive motor intent may be sufficient (Hirsch and Bishop, 1981; Herrero et al., 2018). Furthermore, along with pulmonary afferents, sensory elements mediating effects of slow, deep breathing likely encompass upper airway afferents including those in the nose, pharynx and larynx. Since breathing practices often involve large volume inhalation through the nose with slow controlled exhalation through the mouth, sensory afferent input from these pathways may essentially guide cognitive centers during breathing exercises. Sensorimotor systems of the mouth are likely also integrated to provide an expiratory resistance controlling the rate of airflow from the lungs. Although the contributions of these different sensory systems to the cognitive effects of slow, deep breathing remain to be elucidated, here we primarily focus on contributions from vagal pulmonary afferents and olfactory afferents that have recently been implicated in the context of nasal slow breathing.

### Why Is Slow, Deep Breathing Important?

In the broadest sense, slow, deep breaths are those occurring more slowly than the typical rate of 12–15 breaths per minute in normal adults. Several yogic techniques target the specific frequency of six breaths per minute (0.1 Hz). This frequency maximizes heart rate variability (HRV) (Eckberg, 1983) and dramatically enhances cardiorespiratory synchronization (Peng et al., 2004), the phenomenon whereby the heart rate increases during inhalation and decreases during exhalation. This phenomenon is known clinically as respiratory sinus arrhythmia (RSA), and is greatly exaggerated at slower respiratory frequencies as the difference between peak and trough heart rates is maximized (Bertram et al., 1998; Julien, 2006; Elghozi and Julien, 2007). Peak-to-trough differences in heart rate are the simplest measure of HRV; spectral analysis of heart rate variations based on ECG recordings is another commonly used quantitative method to calculate HRV and reflects the same trend. Reduced HRV is a clinically relevant feature of stress-related disorders including anxiety, depression, and epilepsy (Carney et al., 1995b; Friedman and Thayer, 1998; Brown and Gerbarg, 2005; Mativo et al., 2010).

Respiratory ventilation consists of two components: tidal volume – the volume of air displaced during each breath – and breathing frequency, or ‘respiratory rate.’ Under resting conditions, these two parameters vary inversely in order to satisfy relatively stable metabolic demands for oxygen (O_2_) consumption. Therefore, slower breaths are also necessarily deeper. From here on we occasionally use the terms ‘slow’ and ‘deep’ interchangeably, assuming consistent ventilation to fulfill relatively stable metabolic needs. Tidal volume and respiratory rate are known to vary with behavioral and emotional state. For example, a restful state may be characterized by high tidal volume-low respiratory rate, whereas low tidal volume-high respiratory rate is a characteristic feature of an anxious state (Carnevali et al., 2013). Similarly, respiratory rate increases during stress and is reduced during restful slow wave sleep (Pappenheimer, 1977; Suess et al., 1980). High basal respiratory rates also predict negative cardiopulmonary outcomes (Fieselmann et al., 1993; Hodgetts et al., 2002). This suggests that basal respiratory rate is an index of stress and may reflect its detrimental impact on physiology and behavior.

Slow breathing exercises such as those in pranayama yoga improve stress-related physiology, including autonomic imbalance, cardiopulmonary and neuroendocrine function, and mood (Brown and Gerbarg, 2005; Jerath et al., 2006; Kaushik et al., 2006; Courtney, 2009; Pramanik et al., 2009; Noble et al., 2017a). These techniques can increase HRV by a factor of two or more (Elliott and Edmonson, 2005) and drastically reduce self-reported depression (Janakiramaiah et al., 1998; Lehrer et al., 1999). Such physiological and behavioral benefits are believed to be associated with a shift away from sympathetic dominance toward a net increase in parasympathetic (vagal) tone, thereby reducing stress effects (Brown and Gerbarg, 2005; Jerath et al., 2006). The adaptive changes in autonomic function associated with sustained slow breathing are consistent with the ‘relaxation response’ (Bernardi et al., 1998; Spicuzza et al., 2000; Joseph et al., 2005; Pramanik et al., 2009), a state of deep rest that changes the physical and emotional responses to stressors (Benson et al., 1974).

### Human Subjects Research on Slow, Deep Breathing

Despite evidence of slow, deep breathing’s therapeutic benefit in disorders of autonomic imbalance (Spicuzza et al., 2000; Bernardi et al., 2002; Brown and Gerbarg, 2005; Joseph et al., 2005; Jerath et al., 2006; Kaushik et al., 2006; Courtney, 2009; Pramanik et al., 2009), very few studies have focused on isolating slow breathing from attentional or emotional regulatory elements of training (Ospina et al., 2007). A recent systematic review of slow breathing techniques analyzed the results of 15 studies, concluding that despite some disparities, breath control at low frequencies (<10 breaths/minute) results in decreased anxiety and arousal and increased relaxation (Zaccaro et al., 2018). However, very few studies have shed light on underlying mechanisms or neural substrates. One recent neuroimaging study found that breath-by-breath increases in tidal volume enhanced activity within a dorsal medullary region encompassing the nucleus of the solitary tract (NTS) (Critchley et al., 2015). Another study has found that two distinct meditative practices induce cardiorespiratory synchronization at a respiratory frequency of 0.1 Hz (Peng et al., 2004). Even inexperienced Zen meditators ‘lock in’ at 0.1 Hz (Cysarz and Bussing, 2005). Intriguingly, religious and artistic practices can also facilitate 0.1 Hz cardiorespiratory synchronization, and may have evolved to promote a feeling of wellness that individuals would associate with such practices. For example, the timing of repetition of the Ave Maria in rosary prayer and of yoga mantras cycles at ∼0.1 Hz and produces cardiorespiratory synchronization (Bernardi et al., 2001). Certain forms of rhythmic poetry recitation (e.g., hexameter verse from ancient Greek literature) similarly lead to this phase-locking (Cysarz et al., 2004), as do specific music phrases, as in Verdi’s famous arias (Bernardi et al., 2009). In sum, respiration rate and depth (tidal volume) may covertly control our state of well-being, as the few studies that have investigated the impact of specific respiratory frequencies have found an impressive synchronization of systemic function at the characteristic frequency of 0.1 Hz.

Slow breathing exercises have been proposed as novel therapeutics in a variety of disease states, such as in epilepsy (Yuen and Sander, 2010), where stress is the most common trigger of seizures (Sawyer and Escayg, 2010) and there are measurable changes in autonomic balance (Novak et al., 1999; Mativo et al., 2010). Biofeedback approaches have also been devised to increase RSA (Vaschillo et al., 2002; Song and Lehrer, 2003; Yasuma and Hayano, 2004), a key cardiorespiratory variable enhanced during slow breathing techniques. These approaches, aiming to mimic the heightened cardiorespiratory synchronization achieved during 0.1 Hz breathing and ranging from wearable sensors and patches to mobile phone applications, have proven effective in disorders as diverse as asthma, PTSD, and depression (Lehrer et al., 2004; Karavidas et al., 2007; Zucker et al., 2009).

## The Basic Neurophysiology of Slow, Deep Breathing

**FIGURE 1:**
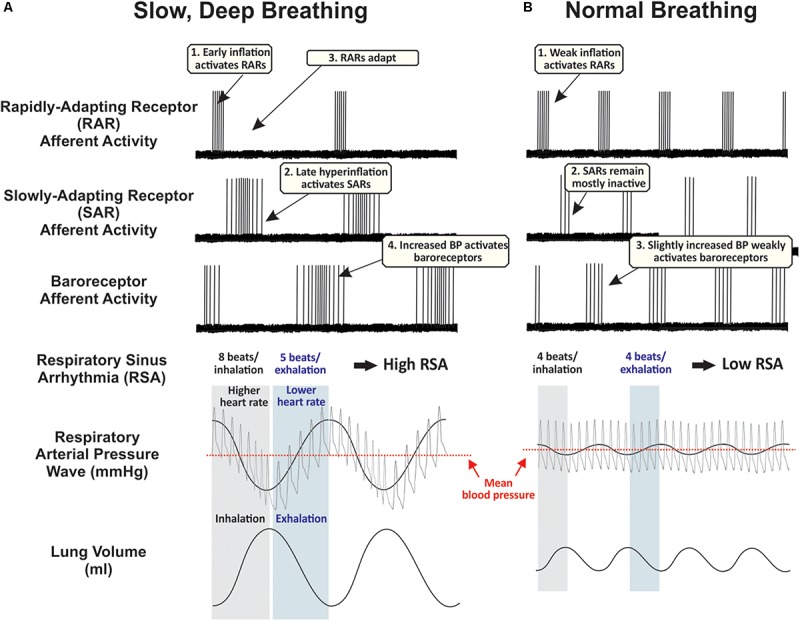
Proposed time course of peripheral afferent effects during **(A)** slow, deep breaths, and **(B)** normal breaths. These hypothetical traces are partly based on records from supportive experiments in anesthetized rats (Ho et al., 2001; Schelegle, 2003). During normal breathing, rapidly-adapting receptors (RARs) are phasically active during inspiration, slowly-adapting receptors (SARs) remain mostly inactive, and baroreceptors are weakly activated during exhalation. During repetitive deep breathing, RARs are activated during the early component of inhalation and then lungs hyperinflate at sufficient pressures to activate low- and high-threshold SARs, increasing traffic through the vagus nerve. Deep exhalations amplify respiratory arterial pressure waves to strongly activate peripheral baroreceptors. Human carotid baroreceptors are slowly-adapting (Eckberg, 1977). Along with these cardiorespiratory vagal afferents, additional airway afferents probably play a significant role in mediating the sensory element of slow, deep breathing, while sensory and motor systems are highly integrated to control the rate of airflow from the lungs and implement cognitive control of respiratory motor drive (see Introduction). BP, blood pressure.

**FIGURE 2:**
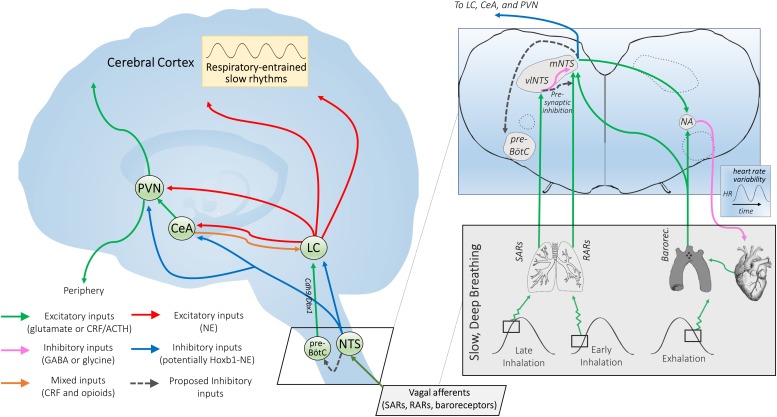
Proposed mechanisms of NTS-mediated relaxation. *Inset*: Slowly-adapting pulmonary receptor (SAR) and rapidly-adapting pulmonary receptor (RAR) afferents project to the brainstem at the level of the medulla and innervate the parasympathetic relay nucleus, i.e., the nucleus of the solitary tract (NTS). That SARs project to a distinct anatomical NTS subregion from RARs and baroreceptors and are associated with a differentiable respiratory phenotype (slow and deep) is consistent with SARs serving a distinct function via a state transition in brainstem autonomic signaling. SARs project primarily to GABAergic inhibitory neurons in the ventrolateral subregion of the NTS (vlNTS) (Berger and Dick, 1987; Kubin et al., 2006), while RARs provide input to the more medial and caudal regions of the NTS (mNTS) (Kubin et al., 2006) to recruit noradrenergic output pathways. Aortic arch and carotid sinus baroreceptor primary afferents mimic RARs in their NTS termination patterns (Dean and Seagard, 1995), but are activated during exhalation instead of inhalation (Figure 1). Glutamatergic projections from the NTS are poised to regulate nucleus ambiguus (NA) cardiac vagal neurons (Neff et al., 1998). The ipsilateral mNTS is the major brain area sending projections to the cardioinhibitory region of the NA (Stuesse and Fish, 1984); though direct projections from the vlNTS have been observed, these are apparently less numerous and have not been shown for sake of clarity. Note that although projections from baroreceptor afferents to the NTS and the NTS to NA appear to cross contralaterally, this is for simplicity of illustration; the majority of these projections are ipsilateral. NTS projections may also feed in to the pre-Bötzinger complex (pre-BötC), potentially inactivating *Cdh9/Dbx1* pre-BötC neurons that appear to activate the locus coeruleus (LC) via glutamatergic projections (Yackle et al., 2017; Vann et al., 2018), and thereby promoting calming. *Outset*: NTS projections to the central nucleus of the amygdala (CeA), paraventricular nucleus of the hypothalamus (PVN), and LC may link cardiorespiratory afferent activation to the effects of slow, deep breathing on stress reduction and attention. Noradrenergic NTS neurons project to downstream limbic areas, sending branching collaterals to the CeA and PVN (Petrov et al., 1993). Several nuclei in the NTS (along with the CeA) also project to the LC, potentially modulating central norepinephrine release (Van Bockstaele et al., 1996, 1999; Berridge and Waterhouse, 2003). NTS projections may serve to integrate autonomic responses with this circuitry and influence downstream targets of the LC (Van Bockstaele et al., 1999). The LC also provides noradrenergic projections to the CeA and PVN (Petrov et al., 1993), supporting the possibility of further signal integration at these higher-order hubs of the central autonomic network. *Hoxb1* noradrenergic neurons (some of which originate from the NTS) provide a substantial input to the LC and peri-LC dendritic field (Chen et al., 2018) and are one neuronal subpopulation that could potently modulate LC activity, contributing to NTS-mediated relaxation. Interconnectivity between the two arms of this proposed neural pathway could influence peripheral and central release of neuropeptides through the PVN and widespread noradrenergic modulation of forebrain areas through the LC that together may impact arousal and responsiveness to stressors.

It is important to consider slow breathing-induced changes in cardiorespiratory circuit recruitment that could trigger the relaxation response. One intriguing possibility is that a unique profile of sensory afferents activated during deep breathing (Figure 1) projects along specific pathways that optimize patterns of sympathetic and parasympathetic output (Figure 2). Cardiovascular and respiratory afferents activated by breathing are known to project to second-order relay neurons in brainstem autonomic regions, most notably the NTS via the vagus nerve (Malpas, 2002; Carr and Undem, 2003; Kubin et al., 2006). The NTS, a relay station for all vagal afferent input, integrates these signals and sends divergent synaptic connections to a variety of brain regions, especially those involved in central autonomic regulation (Petrov et al., 1993; Marieb et al., 2016; Wehrwein et al., 2016). The central autonomic network is a system of cortical, subcortical, and brainstem structures involved in internal regulation and linked to stress-related disorders, including the amygdala, hypothalamus, NTS, and ventrolateral medulla (Loewy, 1990; Benarroch, 1993). Candidate afferents and projection systems, along with the specific physiological events proposed to link slow breathing to a decreased stress response (relaxation) are described below.

### Inspiratory-Activated Pulmonary Afferents, Baroreflexes, and the NTS

Sustained deep breathing repeatedly activates pulmonary stretch receptors in the lungs and bronchi. Pulmonary stretch receptors are innervated by fast-conducting myelinated afferent fibers sensitive to both the static and dynamic aspects of lung volume and transmural pressure (Kubin et al., 2006). Normal breathing preferentially activates rapidly-adapting receptor afferents (RARs). Deep inhalations also activate slowly-adapting pulmonary receptor afferents (SARs) (Schelegle, 2003; Jerath et al., 2006). SARs are activated by exaggerated stretching of the lungs. SARs end inspiration and facilitate expiration as part of the Hering-Breuer reflex (HBR). However, SARs and the HBR may not play a role in setting typical eupneic breathing patterns in adults including in humans (Schelegle, 2003; Dutschmann et al., 2014). Unusually large transpulmonary pressures, such as those achieved during deep breathing, may be required to activate SAR pathways through the NTS during adulthood (Schelegle, 2003). While the reflex circuit mediating the HBR to prevent lung over-inflation (via the vagus to inhibit central inspiratory drive) is fairly well characterized (West, 1995), our model for breathing-dependent relaxation proposes a physiological purpose for SAR pathways through the NTS with anatomical and functional connectivity to central autonomic networks. The HBR may operate during more extreme inspiratory depths to ensure their constraint within an appropriate physiological range. RARs have phasic responses to large lung inflations and deflations (Kubin et al., 2006), whereas SARs respond to large hyperinflations (e.g., 30 cm H_2_O) throughout the duration of the stimulus (Figure 1) (Carr and Undem, 2003). Therefore, deep breaths may preferentially engage SAR-dependent pathways by effectively recruiting both low- and high-threshold SARs (Schelegle and Green, 2001) that remain active throughout the extended inspiratory phase of slow breathing. In contrast, RARs would become silent during long inspirations, reducing their excitatory impact on CNS circuitry. Simultaneously, slow and deep breathing leads to cyclic variations in intrathoracic pressure, impacting venous return to the heart, cardiac output, and consequently blood pressure (Malpas, 2002). These effects result in high-amplitude oscillations in arterial blood pressure known as respiratory arterial pressure waves (Figure 1). Stretch-sensitive peripheral baroreceptors located predominantly in the aortic arch and carotid sinus respond to these changes in arterial blood pressure synchronized to the cardiac and respiratory cycle, contributing to blood pressure and heart rate oscillations that have periods from seconds to minutes (Malpas, 2002; Elliott and Edmonson, 2005; Di Rienzo et al., 2009). All of these afferent mechanoreceptor populations send vagal sensory input to the NTS in the brainstem (Figure 2).

Baroreceptor afferent activation typically initiates a negative feedback cycle whose final output is cardiovagal cholinergic efferents from the nucleus ambiguus innervating the sinoatrial node of the heart. In contrast, SARs act on second-order ventrolateral NTS neurons to directly or indirectly inhibit cardioinhibitory neurons of the nucleus ambiguus (Stuesse and Fish, 1984; Neff et al., 1998; Ezure et al., 2002). Through these second-order neurons SARs inhibit parasympathetic vagal outflow to the heart, leading to an increase in heart rate. Conversely, inhibition of SARs following cessation of deep inhalation (Matsumoto et al., 2006) likely results in strong rebound reciprocal changes that accentuate parasympathetic effects during exhalation, an effect that is consistent with SAR excitation evoking a tachycardia that is gradually masked by a reflex bradycardia (Schelegle and Green, 2001). While RAR and baroreceptor afferent projections to the NTS could impinge on multisynaptic central circuits to inhibit the sympathetic nervous system, SAR pathways would instead activate sympathetic outflow during inhalation. This contradicts the notion of slow breathing as a purely parasympathetic state and suggests the importance of autonomic patterning with the breathing cycle.

### Vagal Tone, Resonance, and Cardiorespiratory Synchronization

#### Vagal Tone and Afferent-Mediated Presynaptic Inhibition

One powerful way that deep breathing could dampen stress-related hyperactivity of the central autonomic circuitry is through vagal modulation. Increased vagal tone is necessary for cardiovascular recovery from psychological stress, while decreased vagal reflexes have been associated with increased risk of cardiovascular disease and death after myocardial infarction (Brown and Gerbarg, 2005). Activating cardiorespiratory vagal afferents – including SARs and baroreceptors – could inhibit CNS activity through at least two distinct mechanisms:

(i) SARs synapse with GABAergic interneurons that *postsynaptically* inhibit third-order NTS neurons projecting to the amygdala, hypothalamus, and other downstream targets (Figure 2);
(ii) Cardiorespiratory vagal afferents could *presynaptically* inhibit local excitatory input to the NTS via primary afferent depolarization (Rudomin, 1967), initiating widespread central autonomic circuitry inhibition.

Presynaptic inhibition is “more powerful than postsynaptic inhibition in depressing the central excitatory actions of almost all primary afferent fibers” (Eccles, 1964). Despite its important role in the CNS, primary afferent presynaptic inhibition has rarely been studied in cardiorespiratory vagal afferents (Rudomin, 1967). All low-threshold cardiorespiratory afferents from this study produced presynaptic inhibition of respiratory afferents, with the magnitude of inhibition increasing with increasing intratracheal pressure. Because both low- and high-threshold SARs respond preferentially to the inspiratory phase of deep breaths (Schelegle and Green, 2001), while peripheral baroreceptors respond to the expiratory phase, presynaptic inhibition may be a physiologically effective means of alternating between sympathetic and parasympathetic afferent drive throughout the ventilatory cycle. Supporting this proposal, afferent stimulation evoked a decrease in blood pressure that was closely correlated with the magnitude of presynaptic inhibition (Rudomin, 1967).

#### Generation of HRV and Mayer Waves

Arterial blood pressure varies during respiratory cycles as a consequence of changing intrathoracic pressures (Malpas, 2002; Figure 1). Baroreceptor afferents detect elevated blood pressure during exhalation and, through direct projections to several subregions of the NTS known to innervate cardioinhibitory regions of the nucleus ambiguus (Ciriello, 1983; Housley et al., 1987), stimulate parasympathetic outflow to the heart to lower blood pressure (Eckberg et al., 1980; Ciriello, 1983; Housley et al., 1987; Schelegle, 2003); reduced baroreceptor activation during inhalation exerts opposite effects. Baroreceptor input has a strong and powerful effect on HRV with each cycle of the breath via these effects on cardiovagal parasympathetic output (Malpas, 2002; Elliott and Edmonson, 2005). Therefore, at lower respiratory frequencies and for deeper breaths, changes in blood pressure will greatly accentuate changes in heart rate, leading to a wide range of observed heart rate values during respiration and consequently amplifying HRV. The arterial baroreflex exhibits positive feedback properties at a frequency of 0.1 Hz (0.4 Hz in rats and mice) (Bertram et al., 1998; Elliott and Edmonson, 2005), resulting in self-sustained oscillations in arterial blood pressure called ‘Mayer waves’ (Julien, 2006), and leading to high-amplitude, vagally mediated heart rate oscillations at 0.1 Hz (Elghozi and Julien, 2007). As elaborated below, voluntarily breathing at a frequency around 0.1 Hz (six breaths per minute) could entrain blood pressure and heart rate to this same resonant frequency. Both the frequency of Mayer waves and the respiratory rate producing maximal HRV are surprisingly invariable within a given species.

#### Multi-System Resonance at 0.1 Hz

All systems have at least one preferred frequency in which oscillations occur at a greater amplitude. These are their resonant frequencies, and independent systems with common resonant frequencies can be mutually reinforcing when coupled. Biologically speaking, resonance is the most energetically and thus metabolically efficient way to increase signal power. In this manner, correlated oscillations of cardiac and respiratory rhythms at one frequency (referred to as their coherence) can lead to mutually reinforcing signal amplification. While the origin of Mayer waves remains incompletely understood, baroreceptor resonance patterns are known to contribute (Julien, 2006). Studies using baroreceptor denervation or blockage with alpha-adrenergic agents (Cevese et al., 2001) implicate peripheral baroreceptors in the corresponding 0.1 Hz rhythm. It is possible that an intrinsic central oscillator in the caudal medulla closely interacts with baroreceptor and pulmonary input in the NTS to change HRV and alter autonomic activity (Julien, 2006). This leads to the idea that breathing at six breaths per minute may maximize HRV by promoting baroreceptor resonance effects to enhance inherent physiological rhythms. The end result would be increased cardiorespiratory synchronization and, via baroreceptor cardiovagal signaling pathways, shifted autonomic balance in the parasympathetic direction.

#### Breath Synchronizes Heart Rate: Respiratory Sinus Arrhythmia (RSA)

Several distinct meditation and yoga techniques profoundly enhance cardiorespiratory synchronization around the characteristic frequency of 0.1 Hz (Eckberg, 1983; Peng et al., 2004; Cysarz and Bussing, 2005). This entrainment of cardiac variability to the respiratory rhythm – so that heart rate increases during inhalation and decreases during exhalation – is referred to clinically as RSA, and is greatly exaggerated at slower respiratory frequencies, such that incremental reductions in respiratory rate result in non-linear increases in RSA (Eckberg, 1983; Rubini et al., 1993; Song and Lehrer, 2003; Pereda et al., 2005). This suggests that a lower respiratory rate will lead to a corresponding increase in RSA, regardless of whether it is possible to reach the frequencies corresponding to peak RSA.

Central drive from the respiratory central pattern generator (CPG) exerts an important influence on the heart and thus RSA (Eckberg, 2003), and recent evidence suggests that the core respiratory CPG is embedded within an anatomically distributed pattern-generating network including the NTS (Dhingra et al., 2019). Pulmonary afferent pathways through the NTS are likely one of several important factors contributing to the increase in cardiac oscillation amplitude (visualized by RSA) at the breathing frequency of 0.1 Hz, although their role has been assigned varying degrees of importance ranging from minor (Koh et al., 1998) to obligatory (Taha et al., 1995). The primary factor appears to be the synchronization of respiratory and cardiac oscillations without any delay (unlike during normal breathing), and out of phase by 180° with blood pressure, allowing the baroreflex to be ideally activated (Laude et al., 1993; Lehrer and Gevirtz, 2014). The presence of Mayer waves at 0.1 Hz also reflects baroreceptor resonance effects at this same frequency that contribute to the increase in RSA amplitude. Thus, the powerful modulation of RSA observed during slow and deep breathing is a complex and multifaceted phenomenon that remains only partially understood.

RSA appears to be a reliable physiological index of respiratory rate’s impact on autonomic function. It is decreased in individuals with depression, anxiety, and panic disorder, and enhanced in physically active individuals (Carney et al., 1995a; Davy et al., 1996; Beauchaine, 2001). Several therapeutically relevant meditation techniques that involve slowed breathing also enhance RSA (Lehrer et al., 1999; Peng et al., 2004; Cysarz and Bussing, 2005), and increases are associated with improved parasympathetic function and wellness (Brown and Gerbarg, 2005). In analogy with repeated exercise promoting physical fitness, voluntary and repeated lowering of respiratory rate may lead to autonomic fitness and promote well-being.

#### Ventilation-Perfusion Matching and Metabolic Benefits

Cardiorespiratory synchronization around 0.1 Hz may enhance ventilation-perfusion matching (Malpas, 2002; Yasuma and Hayano, 2004; Thayer and Sternberg, 2006; Russo et al., 2017). During inhalation, expansion of the chest cavity and the resulting intrathoracic negative pressure increase leads to expansion of blood vessel diameter, effectively increasing perfusion of freshly inhaled O_2_ to vital tissues. Conversely, exhalation corresponds to the ebb in perfusion. Studies have confirmed that pulmonary gas exchange is improved with enhanced RSA, supporting an active physiological role (Yasuma and Hayano, 2004). The net result is decreased energy expenditure (eliminating unnecessary heartbeats during exhalation) and increased metabolic efficiency. This is in line with a study of the relaxation response that found associated changes in systemic gene expression, particularly in markers of primary metabolism and cellular stress responses (Dusek et al., 2008).

## Relating the Basic Neurophysiology of Slow, Deep Breathing to Modulation of Known Stress Pathways

In the previous section, we described afferent pathways and physiological processes recruited by slow breathing. Here, we develop a neurophysiological model (Figure 2) that relates afferent systems to brainstem nuclei and central autonomic networks to explain how a state of calm alertness may ensue.

### Modulation of Anxiety State and Central Autonomic Regulation via the Central Nucleus of the Amygdala and Paraventricular Nucleus of the Hypothalamus

As the major relay station for vagal afferents arriving from the periphery, the NTS forms an early part of the central autonomic network (Loewy, 1990; Benarroch, 1993). Deep breathing-induced pulmonary and baroreceptor afferent activity patterns may lead to the recruitment of specific classes of NTS, hypothalamic and amygdalar neurons identifiable by their anatomical topography (see Figure 2). The overall profile of recruitment could represent a “neural signature” for emotional engagement and wellness. Specifically, NTS neurons appear to engage brain circuitry involved in emotion and internal regulation (Thayer and Lane, 2000; Porges, 2007), with particularly dense projections to subcortical regions including the central nucleus of the amygdala (CeA) and paraventricular nucleus of the hypothalamus (PVN) (Riche et al., 1990; Petrov et al., 1993). In animal studies, acute social stress increases Fos expression in the CeA and PVN (Martinez et al., 1998; Dayas et al., 2001), while depressing HRV and increasing circulating catecholamines (Sgoifo et al., 1999). Given the CeA’s key role in activating sympathetic autonomic output pathways and centrally coordinating the fear network (Ressler, 2010), deep breathing may exert its anxiolytic effects by opposing stress-induced recruitment of the CeA. Reduced firing in amygdala neurons could contribute to physiological calming (Austin, 1998). Furthermore, in cats with experimentally elevated blood pressures and respiratory rates, inactivation of the CeA lowers both values (Zhang et al., 1986b). This supports a bidirectional relationship between the amygdala and NTS, with amygdala-associated emotional state driving increases in respiratory rate and respiratory rate in turn driving amygdala activity via NTS projections.

### Modulation of Arousal and Attention via Pre-Bötzinger Complex – Locus Coeruleus Pathways

Emerging evidence suggests that slow breathing may engage distinct neuronal networks from those employed during normal breathing. A collection of neurons in the brainstem known as the pre-Bötzinger complex (pre-BötC) plays an important role in respiratory rhythm generation. Recent studies in the mouse suggest that removal of a small, molecularly identifiable, neuronal subpopulation within the pre-BötC (*Cdh9/Dbx1* pre-BötC neurons) reduces arousal and leads to a calm behavioral phenotype characterized by increased bouts of stillness and alterations in grooming and sniffing, despite leaving normal breathing largely unaltered (Yackle et al., 2017). The excitatory neurons studied were found to directly project to noradrenergic neurons of the locus coeruleus (LC), a brainstem region implicated in arousal, attention, and responsivity to stress. Through its extensive efferent network, the LC potently regulates systemic function and behavioral state (Berridge and Waterhouse, 2003). Therefore, this pathway may represent an important circuit regulating the balance between calm and arousal behaviors (Yackle et al., 2017). Despite the clear evolutionary importance of preparing an organism to deal with conditions evoking rapid or impaired breathing (Yackle et al., 2017), over-activation of this pathway could be detrimental. Via mechanisms described below, slow breathing could optimize circuit function and reduce behavioral arousal. The hypothesis was recently put forth that respiration and attention comprise a coupled system via the LC (Melnychuk et al., 2018), suggesting a dual role for the LC in slow breathing-induced reductions in arousal and enhancement of flexible attentional states.

### Candidate Neuronal Subpopulations for NTS-Mediated Relaxation

A recent paper demonstrated that activation of a functional subpopulation of noradrenergic neurons (*Hoxb1*-NE) prevalent within the A2 cell group – centered within intermediate and caudal levels of the NTS (Rinaman, 2011) – attenuated behavioral responses to acute stress (Chen et al., 2018). Using neuroimaging, the authors found that *Hoxb1*-NE neuron activation reduced neuronal activity in several stress-related brain regions including the amygdala and LC contrary to the generally recognized role of norepinephrine (NE) in stress circuit activation (Bundzikova-Osacka et al., 2015) and well-known arousing effects of widespread NE release from the LC (Berridge and Waterhouse, 2003). These *Hoxb1*-NE neurons provide substantial input to the LC and peri-LC dendritic field (Chen et al., 2018), suggesting they may directly modulate LC activity (Van Bockstaele et al., 1999). Furthermore, medullary *Hoxb1*-NE neurons project to the CeA (Robertson et al., 2016). This suggests that SAR, RAR, and baroreceptor afferent pathways – via a common relay through the NTS subregions housing *Hoxb1*-NE neurons – may regulate a shared NTS outflow, providing inhibitory noradrenergic control of the central autonomic network (Figure 2). As the CeA is the primary output or effector region of the amygdala and regulates the release of cortisol through the PVN (Ressler, 2010), and the noradrenergic projections of the LC swarm the entire forebrain (Berridge and Waterhouse, 2003), coincident modulation of both pathways may powerfully influence behavioral state. During slow breaths, the balance of these effects could lead to a state of alert relaxation.

Pulmonary afferent systems also project via divergent NTS pathways to central respiratory neurons including those in the pre-BötC and Bötzinger (BötC) regions (Kubin et al., 2006). Correspondingly, the inherent rhythmicity of pre-BötC circuits normally requires dynamic adjustment through network interactions, and these excitatory and inhibitory circuits are controlled by convergent inputs, including from the NTS (Richter and Smith, 2014). The combined effects of a tonic excitatory drive to the NTS and activation of RARs during normal breathing could promote basal states of arousal through *Cdh9/Dbx1* pre-BötC-LC arousal pathways. Conversely, via rhythmic activation of SARs, slow and deep breaths could recruit inhibitory NTS circuitry to restrain activation of pre-BötC-LC arousal pathways. However, whether pulmonary afferent and *Cdh9/Dbx1* neuronal circuits are integrated at the level of pre-BötC or represent two alternative pathways (e.g., sensory reflex-dependent vs. central oscillator-derived) for reducing behavioral arousal remains to be determined. In comparison, it is well-established that SARs monosynaptically activate pump (P-) cells that project to medial and commissural NTS subnuclei (Kubin et al., 2006) to inhibit RAR-cells (Ezure and Tanaka, 2000; Ezure et al., 2002). These same medial and commissural NTS subregions provide the majority of its noradrenergic input to the CeA and PVN (Petrov et al., 1993). Therefore, SAR pulmonary afferent input through the NTS, potentially converging with central respiratory networks, could effectively control activation of subsequent circuits such that these pathways are inhibited during inspiration and disinhibited during expiration (Lopes and Palmer, 1976; Russo et al., 2017). Following SAR-mediated inhibition of medial NTS RAR-cells during deep inhalation, reciprocal inhibition and afterhyperpolarization of SARs during prolonged expiration could result in a profound disinhibitory state that recruits efferent NTS circuitry to dampen forebrain activity.

## Linking Slow Breathing to Global Changes in Brain Activity

**FIGURE 3:**
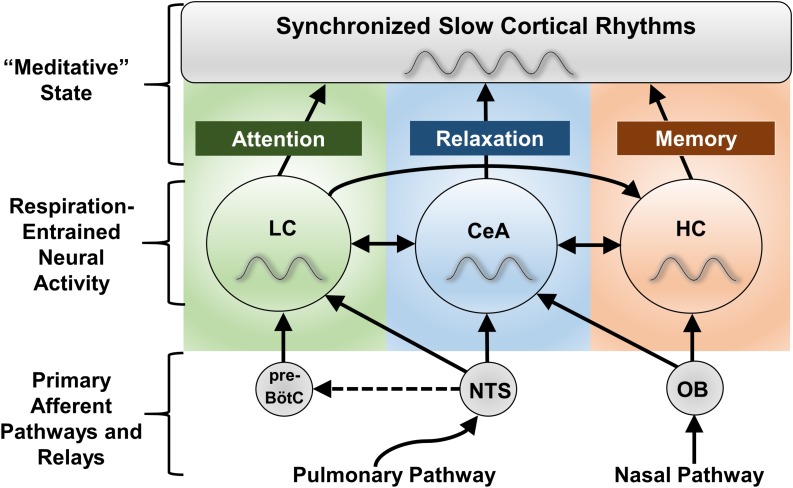
Proposed CNS regions mediating the effects of slow breathing on anxiety, attention, and memory. The locus coeruleus (LC), central nucleus of the amygdala (CeA), and hippocampus (HC) all experience fluctuations in neuronal firing entrained to the respiratory cycle (Zhang et al., 1986a; Chen et al., 1991; Guyenet et al., 1993; Oyamada et al., 1998; Zelano et al., 2016). Despite impacting a broad array of functions, these regions are known to specialize in the regulation of anxiety state (CeA), attention and arousal (LC), and memory (HC), and together project to nearly the entire forebrain. In the forebrain, local processes converge into global respiratory rhythmic slow waves that may serve as the substratum for altered states of consciousness, including those subjectively experienced during meditation techniques involving slow, deep breathing. Interactions between LC, CeA, and HC pathways are proposed to complexly define cognitive and behavioral state. NTS stimulation may also activate the paraventricular nucleus of the hypothalamus (PVN, not shown) through the CeA to regulate the release of cortisol (Ressler, 2010), although this may be an indirect effect (no significant change in hypothalamic activity was observed with stimulation of *Hoxb1* noradrenergic neurons, Chen et al., 2018). Dashed lines indicate hypothetical connections. NTS, nucleus of the solitary tract; OB, olfactory bulb; pre-BötC, pre-Bötzinger complex.

In the Section “Relating the Basic Neurophysiology of Slow, Deep Breathing to Modulation of Known Stress Pathways” we explored the impact of slow breathing on central autonomic regulation and developed a model to account for its effects on stress circuitry. In this section we address whether slow breathing changes higher-order brain activity. Proposed mechanisms include:

(i) Nasopharyngeal breathing activates olfactory afferents that recruit specific *hippocampal rhythms in circuitry* involved in memory processes.
(ii) Cardiorespiratory afferent pathways project *via the NTS to downstream brain regions* including the LC, amygdala and hypothalamus (see previous section; Figure 2).
(iii) Neural activity synchronizes to the respiratory cycle, *entraining slow brain rhythms around 0.1 Hz* throughout stress- and memory-related cortical circuitry (Figure 3).
(iv) Slow brain rhythms *resonate with the default mode network* to promote altered states of consciousness associated with meditation.
(v) Slow breathing drives *mechanical, pulsatile changes in blood and cerebrospinal fluid (CSF) flow* that non-specifically activate mechanosensitive afferent pathways to evoke widespread, global brain synchrony (i.e., broad recruitment of neural circuits).

We provide further detail elaborating on these possibilities below.

### Nasal Respiration Recruits Slow Brain Rhythms to Enhance Memory Function

Slow nasal respiration and slow cortical rhythms can also enhance memory (Marshall et al., 2006; Arshamian et al., 2018), and these effects are thought to be mediated via olfactory bulb-driven neural oscillations that travel to the piriform cortex and onward to the hippocampus (Arshamian et al., 2018). Indeed, nasal respiration entrains hippocampal oscillatory activity and enhances memory performance in humans (Zelano et al., 2016), and the modulatory effects of hippocampal rhythms may coordinate with thalamic and cortical activity (Zaccaro et al., 2018). Respiration could thereby initiate neural activity patterns that reverberate to the whole brain (Piarulli et al., 2018), seen as respiratory rhythmic slow waves (Fontanini and Bower, 2006). It remains unknown whether the widespread prevalence of these slow waves reflects conventional network transmission or is due to volume transmission (Corcoran et al., 2018; Tort et al., 2018b); the latter effect may implicate nitric oxide signaling (Kim et al., 2005). Recent studies in rodents (Lockmann et al., 2016; Biskamp et al., 2017; Heck et al., 2017; Corcoran et al., 2018; Kocsis et al., 2018; Tort et al., 2018a, b) and humans (Zelano et al., 2016; Herrero et al., 2018; Piarulli et al., 2018) have directly linked slow nasal respiration to slow brain rhythms. In rodents these are dissociable from other low-frequency rhythms (Lockmann et al., 2016; Tort et al., 2018b), and modulate local gamma activity in the medial prefrontal cortex (Tort et al., 2018a), organizing prefrontal network activity (Biskamp et al., 2017).

### Full Cortical Synchrony May Emerge From the Collective Effects of Slow, Deep Breathing on Stress- and Memory-Related Circuitry

We have outlined basic physiological changes linking 0.1 Hz breathing to reduced stress circuit activation, examined how slow nasal breathing confers selective benefits on memory circuits, and summarized recent research linking these changes to respiratory-entrained slow brain rhythms. These combined changes could account for a global, ‘meditative’ state characterized by cognitive and behavioral relaxation, alertness, and improved memory. Proposed neural signatures for voluntary slow breathing’s diverse effects are shown in Figure 3.

Nasal pathways through the olfactory bulb and SAR pathways through the NTS may represent two separate mechanisms for the generation of slow cortical rhythms. The combined activity of both could be required for full cortical synchrony. For instance, tracheotomy can weaken respiratory-related oscillations localized to the hippocampus and olfactory cortex in rodents (Fontanini et al., 2003; Lockmann et al., 2016). Comparison of functional cortical differences in response to nasal vs. oral breath pacing in humans could be studied using electroencephalography (EEG). Mechanistically, we hypothesize that the vagal afferent activity induced by slow breathing could entrain EEG activity (Kamei et al., 2000; Busek and Kemlink, 2005) and recruit slow cortical rhythms. Neuronal oscillations in the amygdala and LC have not been studied to the same extent as neocortical and hippocampal oscillations (Pare et al., 2002; Eschenko et al., 2012). However, slow, respiratory-coupled oscillations have recently been discovered in the CeA of mice (Tort et al., 2018b), with human studies further demonstrating respiratory-based entrainment of amygdala circuits (Zelano et al., 2016; Herrero et al., 2018). These phenomena may have previously escaped detection due to the absence of respiration monitoring or similarity with other brain oscillations (Tort et al., 2018b). In rats respiration entrains rhythmic LC activity (Chen et al., 1991; Guyenet et al., 1993; Oyamada et al., 1998), which can form temporary resonant states with frontal cortical oscillatory activity (Lestienne et al., 1997). Moreover, firing of LC neurons presumably provides neuromodulatory input that contributes to EEG slow waves during sleep, to promote plasticity within these circuits (Eschenko et al., 2012).

### Slow Cortical Rhythms Around 0.1 Hz Could Synchronize With Default Mode Network Activity to Promote Meditative States

The impact of slow breathing on cortical rhythms does not appear restricted to 0.1 Hz, as both spontaneous breathing and respiratory-entrained neural oscillations typically occur at frequencies > 0.1 Hz (Bond et al., 1973; Douglas et al., 1982; Krieger et al., 1990; Zelano et al., 2016; Herrero et al., 2018). Intracranial EEG recordings from human epilepsy patients have revealed that cortical oscillatory activity entrains with respiration at slightly higher frequencies (0.16–0.33 Hz) (Zelano et al., 2016; Herrero et al., 2018), with synchronized electrical activity observed in limbic-related brain areas, including the amygdala and hippocampus. Nonetheless, the unique frequency of 0.1 Hz or its harmonics could be crucial for realizing the full cognitive benefits of slow breathing. For instance, a recent study in humans found that mechanically simulating breathing at 0.05 Hz – a subharmonic of 0.1 Hz – slowed cerebral rhythms, enhancing delta-theta EEG activity throughout default mode network (DMN) structures and inducing altered states of consciousness akin to respiration-based meditative practices (Piarulli et al., 2018).

The DMN is a network of brain regions that show highly correlated activity during rest and reduced activation during attention-demanding tasks (Raichle et al., 2001), and contains the medial prefrontal and posterior cingulate cortices (both known to be involved in central autonomic regulation) as core regions (Bär et al., 2015). The DMN is characterized by blood-oxygen-level-dependent (BOLD) fMRI signal fluctuations around 0.1 Hz (Mantini et al., 2007; Jerath et al., 2012; Rayshubskiy et al., 2014; Noordmans et al., 2018). Although the DMN average frequency of 0.1 Hz synchronizes activity in widespread regions of the brain and is highly correlated with respiration (Birn et al., 2008; Jerath et al., 2012), the precise impact of respiratory fluctuations on the DMN is poorly understood (Jerath and Crawford, 2015) and large amplitude 0.1 Hz hemodynamic oscillations observed in human fMRIs indicative of cortical resting states are rarely considered (Rayshubskiy et al., 2014). It has been hypothesized that, via neural activity oscillations around 0.1 Hz possibly attributed to SARs (Jerath et al., 2012), respiratory impulses during slow breathing may synchronize with the DMN (Zaccaro et al., 2018). Since slow, local propagating hemodynamic waves appear related to vasomotion and perfusion of active functional areas (Aalkjaer et al., 2011), respiratory-linked neuronal oscillations around 0.1 Hz could couple the demands of neural activity to perfusion and improve brain tissue capacity (Noordmans et al., 2018), enhancing physiological function. Studies investigating the link between respiration and brain activity have suggested that respiratory rhythms may be an organizing principle of cortical oscillations in the human brain (Herrero et al., 2018). Indeed, slow inhalations at 0.1 Hz have been found to increase EEG spectral power in the delta band and may enhance synchronization in thalamocortical circuits (Busek and Kemlink, 2005). The DMN in turn could support higher processing associated with awareness, memory, and cognitive performance, and may be involved in consciousness (Jerath and Crawford, 2015). Future studies should determine the contributions of respiration to these phenomena and test nasal vs. oral breathing, as well as different rates and modalities of paced breathing or mechanical stimulation, to determine whether 0.1 Hz optimally tunes physiological function.

### Slow Breathing May Entrain Oscillations in Cerebral Blood Flow and Cerebrospinal Fluid for Large-Scale Modulation of Cortical Circuits

While slow wave oscillations have been linked to the respiratory rhythm (Fontanini and Bower, 2006), there is also compelling evidence of cerebral blood flow fluctuations associated with cardiovascular variability (Cencetti et al., 1999) and reductions in cerebral blood flow during slow wave sleep (Townsend et al., 1973). Respiratory activity, which is fairly irregular during waking conditions, tends to stabilize during deep sleep when respiratory rate is precisely correlated with heart rate, enhancing cardiorespiratory synchronization (Bond et al., 1973). Recent research has also discovered that inspiration crucially regulates the flow of CSF (Dreha-Kulaczewski et al., 2015). CSF flow is driven by arterial pulsations and may be impaired in disease states (Mestre et al., 2018). This raises the possibility that a traveling fluid wave through the ventricles could activate central mechanoreceptors to complement the direct effects of slow breathing on neural circuits. Future experiments should track CSF flow rate and correlate it with temporal neural activity to validate this proposition, and determine whether 0.1 Hz respiration optimizes the flow of CSF.

## Future Directions and Proposed Studies

We have thus far described the various components of our hypothesis connecting slow breathing to the modulation of afferent systems, central autonomic networks, and cortical pathways. We have discussed several mechanisms linking 0.1 Hz breathing to systemic physiological function including how slow breathing can enhance RSA via increased baroreflex gain and its impact on optimizing ventilation-perfusion matching (Yasuma and Hayano, 2004), and engaging adaptive systems (Porges, 2007) to promote autonomic balance and relaxation. We conclude by discussing the components of our model (Figures 2, 3) that require further testing, with special emphasis on (i) afferent pathways and proposed neurocircuitry, and (ii) respiration-entrained slow cortical rhythms. Basic mechanistic research will help identify new therapeutic targets, while clinical studies should optimize respiratory strategies for different patient populations, in order to fully unlock the potential benefits of slow breathing.

### Translational Studies Using Animal Models to Assess Underlying Mechanisms

Whether animal studies on vagal afferent activity patterns generalize to human slow, deep breathing practices remains uncertain and in need of further study, although there is evidence of similar pulmonary reflex mechanisms (Kubin et al., 2006). The subsection “Human Subjects Research on Slow, Deep Breathing” summarized recent human studies on 0.1 Hz respiration. An important next step is novel approaches using animal models and/or targeted respiratory feedback. Clinical studies have had difficulty ruling out extraneous variables (e.g., placebo effects and expectancy biases developed during training) as contributors to observed changes, and formulating proper control groups (Ospina et al., 2007). An intriguing possibility is the development of animal models that mimic the pure physiology of slow breaths while ignoring additional cognitive components associated with meditation. A review by Ley concluded that “breathing behavior is amenable to the principles of Pavlovian and operant conditioning” (Ley, 1999). Operant conditioning protocols have had some success altering respiration in rats and humans (Gallego et al., 1994; Ley, 1999; Elliott and Izzo, 2006). Our laboratory recently devised a yoked-control operant paradigm to investigate slow breathing as a technique to reduce stress reactivity (Noble et al., 2017a). Rats learned to decrease their resting respiratory rates, an effect that was maintained between sessions and corresponded with a reduction in anxiety-like behavior. Future studies that enhance the ecological validity of operant conditioning procedures could optimize physiological learning (Ramsay and Woods, 2016). Additional studies on controlled animal respiration have employed classical conditioning paradigms (Gallego and Perruchet, 1991; van den Bergh et al., 1995; Nsegbe et al., 1997), or externally induced (“forced”) respiration paradigms using anesthetized and mechanically ventilated preparations, intubated rats, or hypoxic/hypercapnic animals. The application of these approaches in animals to study slow breathing could represent a new frontier in translational research, with animal experiments identifying specific physiological events that link slow breathing to decreased stress responses (relaxation) for subsequent complementary studies in humans that determine the impact of slow breathing as a component of different meditative practices.

### Neurocircuitry of Slow, Deep Breathing

Due to the overwhelming prevalence of stress-related disorders in the general population (e.g., Wiegner et al., 2015) and the ongoing deficiency of technological and pharmacological tools to ameliorate these conditions, there is an urgent need for studies that mechanistically associate slow breathing with relaxation. Studies should test whether activation of pulmonary afferent pathways leads to physiological effects consistent with our proposed model. For example, although the NTS projects extensively to respiratory control centers (Kubin et al., 2006), how exactly this circuitry may influence pre-BötC-LC “calming” pathways (Yackle et al., 2017) is unknown. SAR afferents project to a region of the NTS containing substance P (SP) (Maley, 1996), and terminate on a class of second-order neurons (P-cells) whose projections include the BötC (Ezure and Tanaka, 1996; Ezure et al., 2002), leading to the hypothesis that SAR-activated SPergic P-cells projecting to the BötC may inhibit pre-BötC-LC pathways (Fong and Potts, 2006). Using targeted approaches to selectively control populations of neurons in the NTS or pre-BötC, recently attempted using genetic methods (Yackle et al., 2017), is one elegant technique that could be performed to more definitively ascertain this connection. Also of relevance are the medial and commissural NTS subregions that provide the majority of its noradrenergic input to the CeA and PVN (Petrov et al., 1993). In contrast to the ventrolateral NTS, these subnuclei coexpress neuropeptide Y (NPY) (Maley, 1996), which is known to promote stress coping and resilience (Sah and Geracioti, 2013). A pivotal role for these NTS projections in reducing stress reactivity is suggested by the recent study showing that activation of a small subpopulation of genetically identifiable (*Hoxb1*-NE) noradrenergic neurons originating from multiple regions including the A2 cell region led to attenuated responses to acute stressors (Chen et al., 2018). The anxiolytic effects of *Hoxb1*-NE neuron activation apparently required noradrenergic signaling through α1 and β adrenoceptors. Pharmacological studies could administer noradrenergic agonists and antagonists via local iontophoretic application and examine stress-related physiology at α1- or β-adrenergic receptors in the CeA or PVN. A subset of these calming noradrenergic projections also coexpressed NPY, which the authors concluded may modulate NE transmission to facilitate “anti-stress” responses; the NPY system may represent a promising complementary target for experimental manipulation. Combining such measures with other steps aimed at clarifying biological mechanisms, such as quantifying measures of circuit activation including Fos expression in the CeA, PVN, or NTS (Dean and Seagard, 1995; Martinez et al., 1998; Dayas et al., 2001), will help determine the underlying neurophysiology of slow breaths. These approaches will ideally be combined with others that enable monitoring of behavioral and cardiorespiratory variables (Rubini et al., 1993; Noble et al., 2017b) to provide a more complete understanding of the mechanisms linking slow breathing to relaxation-related outcomes.

### Optimal Parameters of Slow, Deep Breathing

Future studies should also test whether oral breathing has the same effects on relaxation as nasal breathing, and evaluate the mechanisms through which specific breathing patterns around 0.1 Hz (e.g., decreased inhalation/exhalation ratio) confer selective benefits. In healthy adults breathing quietly, the normal inhalation/exhalation ratio is approximately 1:1.2 but this decreases during deep breathing (Ragnarsdottir and Kristinsdottir, 2006), and yogic techniques emphasize a breath pattern of 0.1 Hz with prolonged exhalation (ratio 1:2) (Van Diest et al., 2014). During slow and deep exhalations, when the respiratory gate through the NTS is “opened,” maximal activation of the pathways described above may evoke a state of parasympathetic dominance. Humans breathing at 0.1 Hz with prolonged exhalation display reduced physiological and psychological indices of arousal and report increased relaxation as compared to prolonged inspiration or equal ratio breathing (Cappo and Holmes, 1984; Van Diest et al., 2014). In a similar vein, it will be important to isolate the relative contributions of different afferent populations to the physiological effects of 0.1 Hz breathing. For instance, a recent neuroimaging study of slow breathing (Critchley et al., 2015) found that baroreflex-based cardiovascular responses (blood pressure and HRV) associated with activity within the ventral medulla while increased tidal volume associated with activity in the NTS and dorsal pons (including the LC). Therefore, the effects of high tidal volume breathing on pulmonary afferent activation may be more connected to its impact on modulating arousal, with baroreceptors and associated ventral medullary regions more directly related to cardiorespiratory effects that optimize ventilation to match perfusion [RSA – see subsections “Breath Synchronizes Heart Rate: Respiratory Sinus Arrhythmia (RSA) and Ventilation-Perfusion Matching and Metabolic Benefits”]. Experimental approaches that target specific primary afferent pathways – such as electrophysiological stimulation of vagal pulmonary afferents in animal models – could provide such a distinction.

### Relationship Between Cortical Synchrony, Mayer Waves, and States of Consciousness

The signal coherence provided by cardiorespiratory synchronization around 0.1 Hz could lead to related emergent phenomena. Autonomic neural circuits could entrain cortical activity around 0.1 Hz, generating slow cortical rhythms similar to those seen during deep sleep. Generation of large arterial pressure 0.1 Hz Mayer waves would facilitate tissue perfusion independent of heart function, thereby reducing cardiac burden with consequent reduced metabolic demand, and may support a sense of physical relaxation. The relaxation response would reduce stress reactivity and could facilitate learning and memory.

While slow breathing induces spatiotemporal patterns of brain activity overlapping with the DMN (Birn et al., 2008; Piarulli et al., 2018), certain meditation techniques emphasizing focused attention or effortless awareness, as well as highly pleasant ‘flow’ states, are associated with reductions in DMN activity (Brewer et al., 2011; Garrison et al., 2013; Ulrich et al., 2016; Scheibner et al., 2017). Humans at rest alternate between activation of the DMN and an anticorrelated task-positive network, with an average switching frequency of 0.05–0.1 Hz (Majeed et al., 2011; Vanhaudenhuyse et al., 2011). Respiratory afferent activity patterns recruited by slow breathing could contribute a rhythmic component to ongoing cortical activity, linking cognitive function to the process of breathing (Heck et al., 2017). Studies undertaken in animal and human subjects should further investigate slow breathing for its impact on DMN-associated slow brain rhythms and cognition. Although recent work has helped to clarify the relationship between respiration and slow rhythms in humans (Zelano et al., 2016; Herrero et al., 2018), studies are needed to establish a direct link with breathing around 0.1 Hz. This frequency may maximize resonant effects, possibly directly or via cerebral blood flow fluctuations associated with cardiovascular variability (Cencetti et al., 1999). Slow cortical oscillations observed during sleep or under deep anesthesia occur at approximately the species-specific frequency of Mayer waves in the cat (0.3 Hz) (Steriade et al., 1993), but little research has explored the connection between vasomotion, Mayer waves, and resting state functional connectivity (Bumstead et al., 2017). Slow breathing could enhance inherent slow rhythms while activating a unique profile of neural networks to explain the subjective experience of alert relaxation vs. deep sleep. The possibility that slow oscillations are a Mayer wave-linked phenomenon, with potential links to the DMN (Jerath and Crawford, 2015), should be further examined using a combination of electrophysiological and neuroimaging approaches. Studies in meditators breathing at different rates and using different strategies (e.g., voluntary control vs. passive attention) (Herrero et al., 2018) will help us understand the relationship between these global phenomena and how they might be recruited by targeted breathing practices to engage subjective states of relaxation and awareness.

## Author Contributions

DN drafted the manuscript with SH providing substantial revisions.

## Conflict of Interest Statement

The authors declare that the research was conducted in the absence of any commercial or financial relationships that could be construed as a potential conflict of interest.
